# Constructing the Microbial Association Network from Large-Scale Time Series Data Using Granger Causality

**DOI:** 10.3390/genes10030216

**Published:** 2019-03-14

**Authors:** Dongmei Ai, Xiaoxin Li, Gang Liu, Xiaoyi Liang, Li C. Xia

**Affiliations:** 1 Basic Experimental of Natural Science, University of Science and Technology Beijing, Beijing 100083, China; 2 School of Mathematics and Physics, University of Science and Technology Beijing, Beijing 100083, China; lixiaoxinustb@sina.com (X.L.); s20180807@xs.ustb.edu.cn (G.L.); b1090632112@sina.com (X.L.); 3 Department of Medicine, Stanford University School of Medicine, 269 Campus Dr., Stanford, CA 94305, USA

**Keywords:** Granger causality, conditional Granger causality, microbial association network, time series data, marine microbes

## Abstract

The increasing availability of large-scale time series data allows the inference of microbial community dynamics by association network analysis. However, correlation-based association network analyses are noninformative of causal, mediating and time-dependent relationships between microbial community functional factors. To address this insufficiency, we introduced the Granger causality model to the analysis of a recent marine microbial time series dataset. We systematically constructed a directed acyclic network, representing both internal and external causal relationships among the microbial and environmental factors. We further optimized the network by removing false causal associations using the conditional Granger causality. The final network was visualized as a Granger graph, which was analyzed to identify causal relationships driven by key functional operators in the environment, such as *Gammaproteobacteria*, which was Granger caused by total organic nitrogen and primary production (*p* < 0.05 and Q < 0.05).

## 1. Introduction

Microbial communities involve thousands of interacting species with millions of metabolic and signal transduction relationships [1]. The rapid development of high-throughput sequencing techniques has resulted in a sharp increase of longitudinal molecular ecological studies, which follow the changes of microbial communities in various environments over time [2]. Those studies investigated not only the long-term changes in marine ecosystems [3,4,5], but also those within the human body [6,7]. The molecular time series data generated from such studies has shed light on the dynamics of microbial communities, and continue to expand in scale and complexity [8]. It is thus imperative to develop and apply novel quantitative methods that are efficient, accurate and robust to build predictive models and identify the determinant factors that can cause changes to significantly disrupt or shift the equilibrium of microbial communities [2].

Our understanding of microbial communities has benefited from many association network analysis studies. For instance, Barberan et al. (2013) studied the associations among microbial taxa and applied network analysis to identify the microbial community structure within soil [9]. Faust et al. (2012) created a microbiome-wide interaction network of the human body to determine the co-occurrence and co-exclusion relationships among the microbial taxa across individuals, potentially indicative of their ecological functionality [10]. Xia et al. (2011) extended the LSA [11] algorithm to time series data with replicates and developed the extended LSA (ELSA) algorithm to study the subinterval and time-delayed associations with highly improved efficiency. These earlier studies and many others have revealed many interesting dynamics of the microbial community, such as the typical dominance of positively directed associations within microbial communities and the existence of signature environmental factors as hubs within the microbial community network [12].

However, those existing tools and methods for time series analysis and network inference were based on correlation, or its variants. Since the correlation-based measures are only associative, they can only infer relationships that are non-causal. They cannot be used to determine whether one microbial factor is the determinant of another or whether other mediating factors are present in their association. In this paper, we adapted and applied Granger causality analysis to a marine microbial community time series dataset, and constructed directed networks to identify potentially causal, mediating and time-dependent relationships among all microbial community factors. The developed approach is generally applicable to the network analysis of molecular ecological time series data.

## 2. Materials and Methods

### 2.1. Granger Causality Model

The Granger causality model was first proposed by Granger [13] and systematically reviewed in Geweke [14]. The model was based on autoregressive models [15], and it is valid when the time series being analyzed is stationary, a requirement generally satisfied by the microbial time series data. We denoted two microbial functional factors and their time series data as *X*_1_ and *X*_2_, where $X_{1}=(x_{1}(1),\;x_{1}(2),\;\cdots ,\;x_{1}(t))$, $X_{2}=(x_{2}(1),\;x_{2}(2),\;\cdots ,\;x_{2}(t))$, t=1,2,⋯,T, and *T* is the number of time points. We introduced the p-order single-variable autoregressive model for $X_{1}$:

(1) $$x_{1}(t)=\sum_{k=1}^{p}a_{1}(k)x_{1}(t-k)+u_{1}(t),$$

where in Equation (1), the value at time *t* in time series $X_{1}$ is decomposed as a linear combination of the preceding *p* moments. Similarly, we obtained autoregressive model for $X_{2}$:

(2) $$x_{2}(t)=\sum_{k=1}^{p}a_{2}(k)x_{2}(t-k)+u_{2}(t).$$

Based on this bivariate autoregressive model, the bivariate linear Granger causal model additionally considers the causing effect of $X_{1}$ on $X_{2}$ and $X_{2}$ on $X_{1}$:

(3) $$x_{1}(t)=\sum_{k=1}^{p}a_{1,1}(k)x_{1}(t-k)+\sum_{k=1}^{p}a_{1,2}(k)x_{2}(t-k)+w_{1}(t),$$

(4) $$x_{2}(t)=\sum_{k=1}^{p}a_{2,2}(k)x_{2}(t-k)+\sum_{k=1}^{p}a_{2,1}(k)x_{1}(t-k)+w_{2}(t).$$

In Equations (3) and (4), the value at time *t* in time series of one series is now decomposed as a linear combination of the preceding *p* moments of itself as well as those of the other series.

The effect of Granger causality is measured by information content [16]. In information theory, for variables $X_{1}$ and $X_{2}$, if we use the past information of $X_{1}$ and $X_{2}$ to predict $X_{1}$, then the error can be less than that obtained by using the past information of $X_{1}$ alone. In other words, the past information of $X_{2}$ affects the current value of $X_{1}$ so that $X_{2}$ –> $X_{1}$, or equivalently, $X_{2}$ Granger causes $X_{1}$ [17,18]. One can determine whether $X_{1}$ Granger causes $X_{2}$ by use of the following Granger Causality (GC) statistic:

(5) $$GC_{x_{1}\to x_{2}}=\ln\frac{\mathrm{var}\left(u_{2t}\right)}{\mathrm{var}\left(w_{2t}\right)}.$$

If there is no causal influence from $X_{1}$ to $X_{2}$ then $GC_{x_{1}\to x_{2}}$ = 0, otherwise we have $GC_{x_{1}\to x_{2}}$ > 0.

### 2.2. Statistical Significance of Granger Causality

In the case of bivariate Granger causality model, one can use the F-test to find the statistical significance of the GC statistic with the null hypothesis that there is no causality from $X_{1}$ on $X_{2}$ [18], i.e., $a_{2,1}(k)=0,k=1,\cdots ,p$. The F statistic [19] is calculated as:

(6) $$F_{x1\to x2}^{GC}=\frac{T-2p-1}{p}\frac{RSS_{x_{2}|x_{2}^{-}}-RSS_{x_{2}|x_{2}^{-},x_{1}^{-}}}{RSS_{x_{2}|x_{2}^{-},x_{1}^{-}}},$$

where $RSS_{x_{2}|x_{2}^{-}}$ and $RSS_{x_{2}|x_{2}^{-},x_{1}^{-}}$ are the Residual Sum of Squares of $\sum u_{2}^{2}\left(t\right)$ and $\sum w_{2}^{2}(t)$.

### 2.3. Conditional Granger Causality

However, in a dynamic system involving three or more factors, a bivariate GC model cannot tell whether the causal effect between a pair of time series is caused indirectly by the third time series. For example, consider two causality relationships Y→Z→X and Y→X where Y→Z→X means that Z directly Granger causes X, and Y indirectly Granger causes X, while Y→X means that Y directly Granger causes X. Assume that Y→Z→X is the actual causality relationship. In this case, the bivariate Granger causality model will incorrectly infer that Y→X. To eliminate this type incorrect inference, we apply a conditional Granger causality [18,20] based correction on the obtained bivariate Granger causality associations to remove spurious causalities.

Without the loss of generality, we assume that U is a triplet time series comprised of three factors: $U_{t}=(X_{t},Y_{t},Z_{t})$. To determine if Z explains the Granger causality between Y and X, we build the *p*-order autoregressive models as follows:

(7) $$\begin{array}{l} X_{t}=\sum_{k=1}^{p}A_{xx,k}X_{t-k}+\sum_{k=1}^{p}A_{xy,k}Y_{t-k}+\sum_{k=1}^{p}A_{xz,k}Z_{t-k}+\varepsilon_{x,t}\\ X_{t}=\sum_{k=1}^{p}A_{xx,k}^{\prime }X_{t-k}+\sum_{k=1}^{p}A_{xz,k}^{\prime }Z_{t-k}+\varepsilon_{x,t}^{\prime } \end{array}$$

Equation (7) is similar to Equations (1) and (2), where the only difference is the inclusion of the conditional variable Z [18,20]. Thus, the conditional Granger causality (GC) is calculated as:

(8) $$GC_{Y\to X|Z}=\ln\frac{\mathrm{var}\left(\varepsilon_{x,t}^{\prime }\right)}{\mathrm{var}\left(\varepsilon_{x,t}\right)}.$$

### 2.4. Granger Causality Graph

The graphical representation of causal structures can be traced back to 1972 when Arthur Goldberger introduced path diagrams to linear structural equation systems [21]. In 2003, Dahlhaus and Eichler [22] extended those graphical models to visualize Granger causality between variables. Then, Eichler [23,24] proposed the Granger causality graph and studied its Markovian property. More recently, Wei et al. [25] proposed a method to identify Granger causal structures in multidimensional time series based on information theory.

By applying the bivariate Granger causality and Granger conditional causality models to pairwise factors, we determined if a pairwise causality relationship exists, including its direction. We presented such Granger causality as a directed edge in the Granger causality graph. Our overall algorithm is as follows:

(1) After all pairwise Granger causality tests were done for the system U, we generate all the directed edges of graph $G=(V,E^{d},E^{u})$ according the definition of Granger causality graph [25].
(2) We next optimize the graph $G=(V,E^{d},E^{u})$ using the conditional Granger causality:
  - We identify the set $E_{i\to j}=\{j|(i,j)\in E^{d}or(i,j)\in E^{u}\}$ for all variables $x_{i},i\in V$.
  - We construct the triplet U=[X,Y,Z] $\forall j\in E_{i\to j}$, where $X=x_{i},Y=x_{j},Z={\left\{x_{k}\right\}}_{k\neq i,j}$
  - We perform a conditional Granger causality analysis of U and remove the edge from X to Y if any spurious indirect causality was identified (see Equations (7) and (8)).
(3) We output the updated $G^{\prime }$ with all spurious edges removed, which satisfies the requirements of the standard Granger causality graph [25].

### 2.5. Marine Microbial Time Series Datasets

We used two marine molecular ecological time series datasets, i.e., the San Pedro Ocean Time-Series (SPOT) [26] and the Plymouth Marine Lab (PML) [4] in our analysis. These microbial datasets were downloaded from their published papers [3,4,26]. The SPOT data were generated by the San Pedro Ocean Microbial Observation Station of the University of Southern California in the United States. The samples were collected monthly based on sampling depth, and there were 44 time points. The metagenomics data were generated from samples collected at a depth of 5 m. The missing data were deleted. To reduce the dropout effects owing to high frequency of zero values on the inferred causality network, we removed operational taxonomic units (OTUs), which had a mean of less than 1 and which had zeros in more than half of the total time points.

Before carrying out the Granger causality analysis, we conducted stationary tests on the remaining 127 variables, consisting of 100 OTUs and 27 environmental factors in the SPOT dataset (Supplementary Table S1). We used the augmented Dickey–Fuller (ADF) unit root test to remove the non-stationary series in environmental variables, such as the sample location, temperature, light intensity, which are predominantly cyclical or artificially introduced factors. After the removal, 93 OTUs and five environmental factors remained, including chlorophyll a concentration (ChlA), leucine incorporation (Leu), dissolved nitrate (NO3), dissolved silicate (SiO3), and thymidine incorporation (Thy) (Supplemental Table S2). In the data’s original publication, Steele et al. provided taxonomy names of all SPOT OTUs (Supplementary Materials Table S3). In our results, we supplied the class name of an OUT in brackets.

We also analyzed the PML data from the Western Channel Observatory. This dataset contained 72 instances, which were collected from January 2003 to December 2008 from the Plymouth Station near the English Channel. In addition to the metagenomic data, the dataset recorded environmental factors, such as photosynthetically active radiation, North Atlantic Oscillation data, day length, primary productivity, and temperature. We performed PML data cleaning using the same process we applied to the SPOT data, which left us with 71 variables for analysis, including 58 OTUs and 13 environmental factors (Supplemental Table S4). Using the ADF test, we further removed non-stationary environmental variables. We finally obtained a dataset of 66 variables, consisting of 58 OTUs and eight environmental factors, including day length (Day length), primary production (PP), mixed layer depth (MLD), ammonia (NH4), chlorophyll A (ChlA), NO2_NO3 (NO2 + NO3), silicate (Sil), and total organic nitrogen (TON) (Supplemental Table S5). In the data’s original publication, Gilbert et al. provided detailed taxonomy names of all PML OTUs (Supplementary Materials Table S6).

After the data cleaning, we performed the bivariate Granger causality computation and statistical testing for both the SPOT and PML datasets and constructed their causal association networks (Granger graphs), after removing spurious causal relationships using the conditional Granger causality analysis. Since the pairwise Granger causality testing of all variables constitutes multiple testing, we employed Storey’s Q-value method [27,28] to control the significance level of Granger causality. Statistical significance was determined based on a Q-value <0.05. We used Cytoscape [29] to visualize the final Granger causality graph.

## 3. Results

### 3.1. The Marine Microbial Causality Network Based on SPOT Data

**Figure 1:**
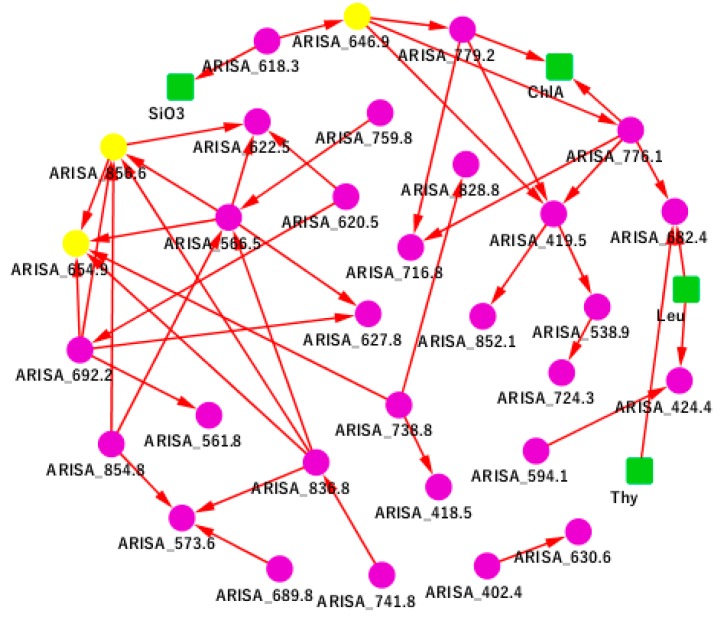
The San Pedro Ocean Time-Series (SPOT) Granger causality network. A green square represents an environmental factor, including ChlA, Leu, SiO3, and Thy. A purple circle represents an operational taxonomic unit (OTU). A yellow circle represents a highlighted key OTU which was discussed in the main text. The directed edge A→B represents A Granger causes B.

**Figure 2:**
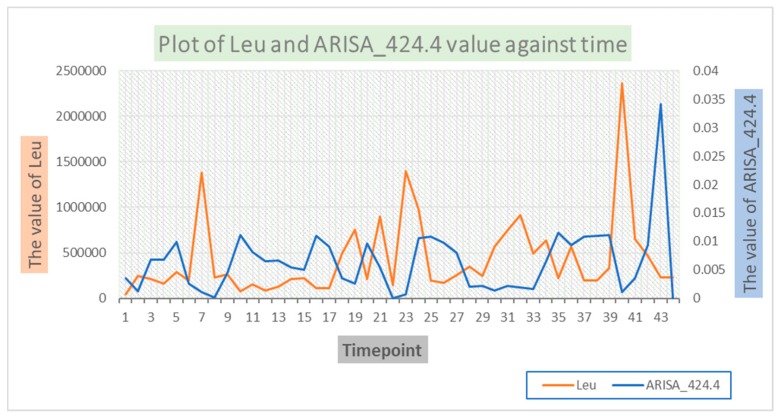
Change of the abundance levels of Leu and ARISA_424.4 over time. It can be seen that the Leu level peaked at the 7th, 18th, 23rd and 40th time points. These peaks were reproduced in the ARISA_424.4 level with a delay at the 10th, 20th, 26th and 42nd time points, respectively. The observed pattern is an intuitive confirmation of the Granger causality from Leu on ARISA_424.4.

We observed a moderate level of causal associations between environmental variables and OTUs, whereas a high level of causal associations among microbial factor themselves. From Figure 1, we can see that Leu Granger causes (we hereafter simply abbreviated “Granger causes” as “causes”) ARISA_424.4 (*Actinobacteria*) and ARISA_682.4 (*Alphaproteobacteria*), meaning that the past Leu levels affect the current value of ARISA_424.4 and ARISA_682.4. The causal effect is obvious when the abundance levels of Leu and ARISA_424.4 were co-plotted along time (Figure 2). As one can see that the Leu level peaked at the 7th, 18th, 23rd and 40th time points. These peaks were reproduced in the ARISA_424.4 level with a delay at the 10th, 20th, 26th and 42nd time points, respectively. This observed pattern is an intuitive confirmation of the Granger causality from Leu on ARISA_424.4. Similarly, we can see Thy causes ARISA_682.4 (*p* = 0.00004-05, Q = 0.004721612).

We also observed that ARISA_618.3 (*Gammaproteobacteria*) causes SiO_3_ (dissolved silicate). According to Fortunato et al., *Gammaproteobacteria*, especially the *Oceanospirillales* family, increased with the salinity, which is supported by our finding here [30]. Similarly, ARISA_776.1 (*Sphingobacteria*) and ARISA_779.2 (*Sphingobacteria*) cause Ch1A. Ch1A represents chlorophyll A concentration, which is a marker chemical for measuring oxygen content in the environment [31]. Meanwhile, the order *Sphingobacteriales* consists mainly of aerobic organisms. Thus, *Sphingobacteriales* may be closely related to oxygen content in the environment. This discovery is important for the further cultivation and research of *Sphingobacteriales*.

### 3.2. The Marine Microbial Causality Network Based on PML Data

**Figure 3:**
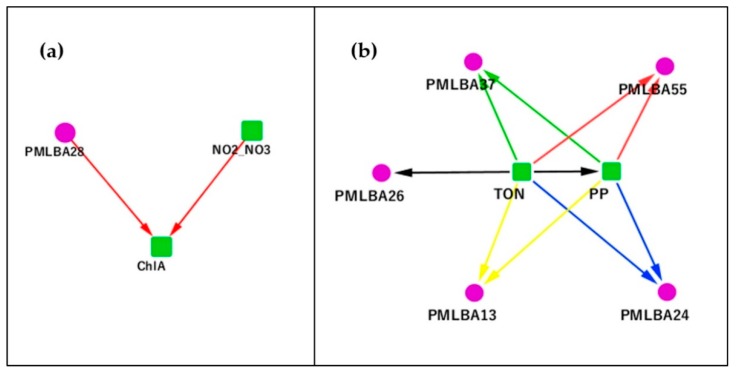
The Granger causality subnetwork of Ch1A and TON-PP of Plymouth Marine Lab (PML) data. A green square represents an environmental factor. A purple circle represents an OTU. (**a**) A subnetwork centered on Ch1A, whose level was only casually affected by the NO2_NO3 and PMLBA28 levels. (**b**) A subnetwork centered on the TON-PP pair, which jointly causally affected four bacterial OTUs.

The analysis of the PML data (see Supplementary Figure S1) revealed more complex causal relationships among microbes as compared to the SPOT data. We noticed that the environmental variables were virtually driving almost all causality relationships in the PML data. In Figure S1, we can see that the core driving factor of the entire PML microbial community is NO2_NO3, which represents the concentrations of nitrite and nitrate. We also found that Ch1A had a unique role in the PML community, which was only affected by the levels of NO2_NO3 and PMLBA28 (Bacteroidetes_03_45) according to the causality network (see Figure 3a).

This relation is confirmed by an independent study, which found a significant positive linear relationship between oxygen and Ch1A(chlorophyll-a concentrations) [32]. However, the previous study failed to determine the direction of causality. Our finding here established the phylum *Bacteroidetes* as a casual and positive regulator of marine ChlA, which might explain its role as a major cellulolytic bacterium [33]. According to the study of Jensen et al. [34], nitrogen fixation on the basis of cellulose requires an anaerobic environment. Thus, the subnetwork in Figure 3a also revealed a symbiotic relationship in which nitrogen-fixing bacteria consume oxygen and use nitrates and nitrites to create a favorable condition for anaerobic cellulose-decomposing microbes.

Biological nitrogen fixation was previously reported as an important source of nitrogen to support oceanic primary production [35,36]. The result was now confirmed by the Granger analysis that TON (total organic nitrogen) causes PP (primary production). In our result (see Figure 3b), the TON-PP pair jointly regulates four other OTUs (PMLBA13, PMLBA24, PMLBA37 and PMLBA55, representing *Bacteroidetes_03_6*, *Bacteroidetes_03_37*, *Bacteroidetes_03_27*, and *Gammaproteobacteria_03_170* respectively). We have applied a conditional Granger causality test to adjust for any indirect effects that may be present in these four triangular network relationships. The computed conditional F-values showed that TON directly Granger causes the other four OTUs. As expected, these nitrogen-fixing *Gammaproteobacteria* are major nitrogenase gene-containing phylotypes in the marine environment, e.g., as suggested in Mellon and Jabir et al. [37,38].

### 3.3. Removing Spurious Causal Relationship Using the Conditional Granger Causality

**Figure 4:**
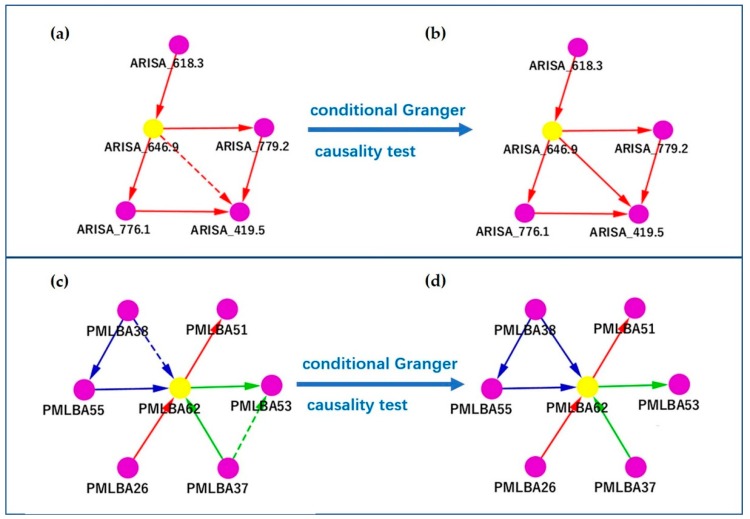
Examples of determining causal relationships using the conditional Granger causality. Shown are subnetworks centered at ARISA_646.9 and PMLBA62 based on SPOT and PML data respectively: (**a**) the subnetwork centered on ARISA_646.9 (before the conditional Granger causality test); (**b**) the subnetwork centered on ARISA_646.9 (after the conditional Granger causality test); (**c**) the subnetwork centered on PMLBA62 (before the conditional Granger causality test); (**d**) the subnetwork centered on PMLBA62 (after the conditional Granger causality test). A green square represents an environmental factor, including ChlA, Leu, SiO3, and Thy. A purple circle represents an OTU. A yellow circle represents a highlighted key OTU which was discussed in the main text.

In many occasions, we found it critical to remove these spurious causal relationships using the conditional Granger causality. Based on the Granger causality network, we used conditional Granger causality to optimize subnetworks and remove spurious causality relationships to find the real causal, mediating and time-dependent relationships between microbial community factors. In Figure 4, we show two such examples arising from the SPOT and PML respectively. In Figure 4a, we show a subnetwork centered at ARISA_646.9, in which the dotted edge between ARISA_646.9 and ARISA_419.5 means that ARISA_646.9 may have a direct causal effect on ARISA_419.5, or an indirect effect, which could potentially be explained by its effect on ARISA_776.1 and ARISA_779.2. The F values calculated by the Equation (8) were F1 = 0.1965 and F2 = 0.1599, which means ARISA_646.9 directly Granger causes ARISA_419.5 and the edge is solid in the final network (Figure 4b). Similarly, the dotted edges in Figure 4c represent potential indirect causes. However, in Figure 4c, F1 = 0.2009, F2 = 0.0102. The cause from PMLBA38 to PMLBA62 was confirmed to be direct, while the one from PMLBA37 to PMLBA53 was confirmed to be indirect and these edges were solidified or removed accordingly in the final network (Figure 4d). There were many similar mediations in the Granger causality network, and its specific biological significance needs further verification.

## 4. Discussion and Conclusions

In this paper, we employed the Granger modeling to perform causality analysis of marine microbial communities in different geographic locations and developed an algorithm to construct the Granger graph using conditional Granger causality test. Before we adapted the Granger causality model to microbial ecological time series data, it has been widely used in economics, neuroscience and other branches of sciences [39,40,41,42]. Especially in neuroimaging and, more broadly, in neurophysiology, G-causality implements a statistical, predictive notion of causality whereby causes precede, and help predict, their effects [39]. However, there are limits in the application of Granger causality. Lionel Barnett et al. proposed that Granger causality estimates may be severely biased or of high variance, and fail to reveal the full structural/causal mechanisms of a system [43]. Therefore, we should use it carefully. Additionally, it should be noted that Granger causality only indicates statistical causality, while the true causal effect has to be validated with controlled experiments.

Our Granger causality networks revealed interesting findings based on these datasets. In the SPOT data, we found that Leu causes ARISA_424.4 and ARISA_682.4 and Thy causes ARISA_682.4. These associations were not identified by Steele et al. in their original paper. However, we found that Thy had fewer connections than Leu, which is the same as the discovery of Steele et al. [26]. Additionally, we found ARISA_618.3 causes SiO3 and ARISA_618.3, which represents the class *Gammaproteobacteria*. In the PML data, we found that TON (total organic nitrogen) causes PP (primary production), where the original study by Gilbert et al. [4] only identified an undirected correlation between the two. Our results supported a conclusion that the biological nitrogen fixation is an important source of nitrogen to support oceanic primary production, which was independently proposed by other reports [35,36] in the literature. In particular, the TON and PP jointly cause several *Bacteroidete* and *Gammaproteobacteria* species.

Finally, applying the conditional Granger causality test effectively could eliminat the indirect causal relationships in a large-scale analysis and help validate real and direct causal relationships. For example, we verified with conditional Granger causality that PMLBA37 does not directly cause PMLBA53. The causal effect was actually mediated by an intermediate factor PMLBA62. Therefore, applying the conditional Granger causality analysis with the original Granger analysis is critical in large-scale network analyses to confirm direct causal effects, as well as to identify indirect effects as mediated by other factors.

## Supplementary Materials

The following are available online at https://www.mdpi.com/2073-4425/10/3/216/s1. Figure S1: Granger causality network for PML data, Table S1: Abundance of SPOT data before ADF testing, Table S2: Abundance of SPOT data after ADF testing, Table S3: Taxonomy name of SPOT data, Table S4: Abundance of PML data before ADF testing, Table S5: Abundance of PML data after ADF testing, Table S6: Taxonomy name of PML data.
