# Supplementary material for: Constructing the Microbial Association Network from Large-Scale Time Series Data Using Granger Causality
Source: Genes (Basel). 2019 Mar 14;10(3):216. doi: 10.3390/genes10030216 (PMC6471626; doi:10.3390/genes10030216)
Supplement: Supplementary file 1 [file genes-10-00216-s001.zip › Supplementary/Supplementary material.docx]

**Supplementary Materials:** Figure S1: Granger causality network for PML data, Table S1: SPOT data on the one-to-one correspondence between OTUs and specific microorganisms, Table S2: PML Data on the one-to-one correspondence between OTUs and specific microorganisms.


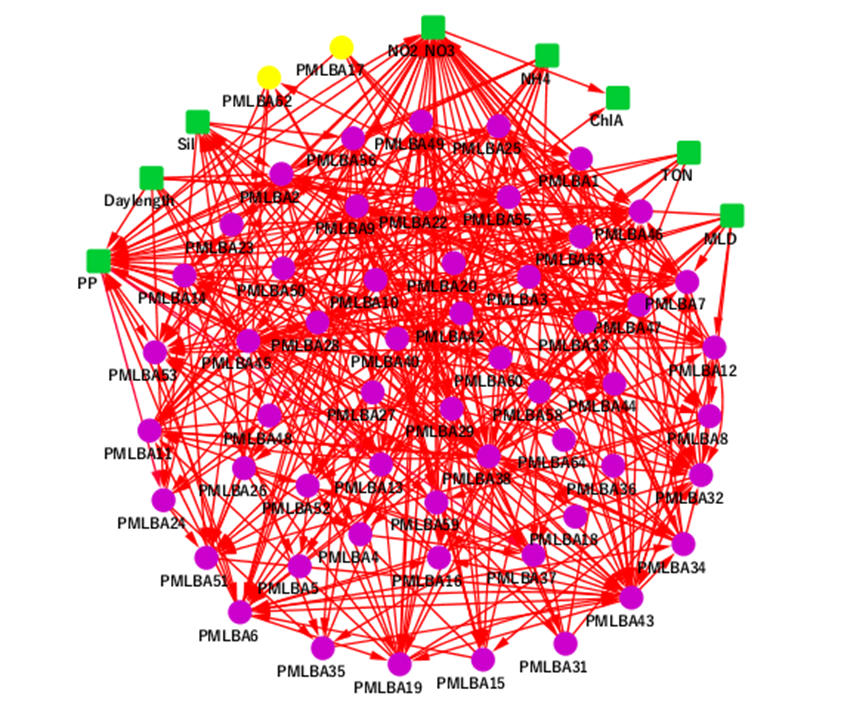


**Figure 1.** Granger causality network for PML data. The green squares represent environmental factors, and the circles with different color represent OTUs. The yellow circles are OTUs of interest in this paper. Those environmental factors included Day length, PP, MLD, NH4, ChlA, NO2_NO3, Sil, and TON.

**Table S1**. Abundance of SPOT data before ADF testing **Table S2**. Abundance of SPOT data after ADF testing **Table S3**. Taxonomy name of SPOT data

**Table S4**. Abundance of PML data before ADF testing **Table S5**. Abundance of PML data after ADF testing

**Table S6**. Taxonomy name of PML data
