# Supplementary figures and images for: Constructing the Microbial Association Network from Large-Scale Time Series Data Using Granger Causality
Source: Genes (Basel). 2019 Mar 14;10(3):216. doi: 10.3390/genes10030216 (PMC6471626; doi:10.3390/genes10030216)

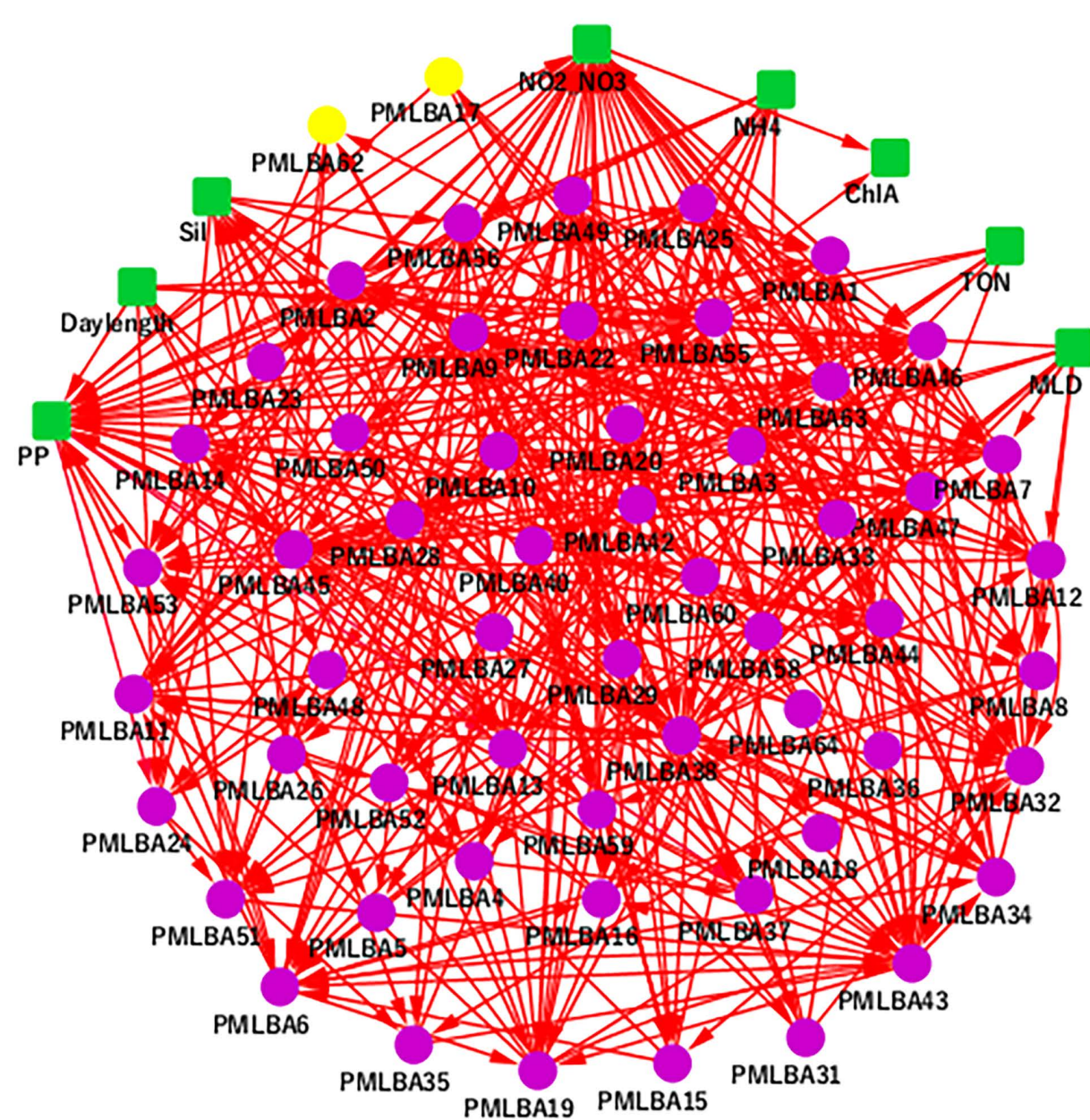

Supplement: Supplementary file 1 [file genes-10-00216-s001.zip › Supplementary/figureS1.pdf]

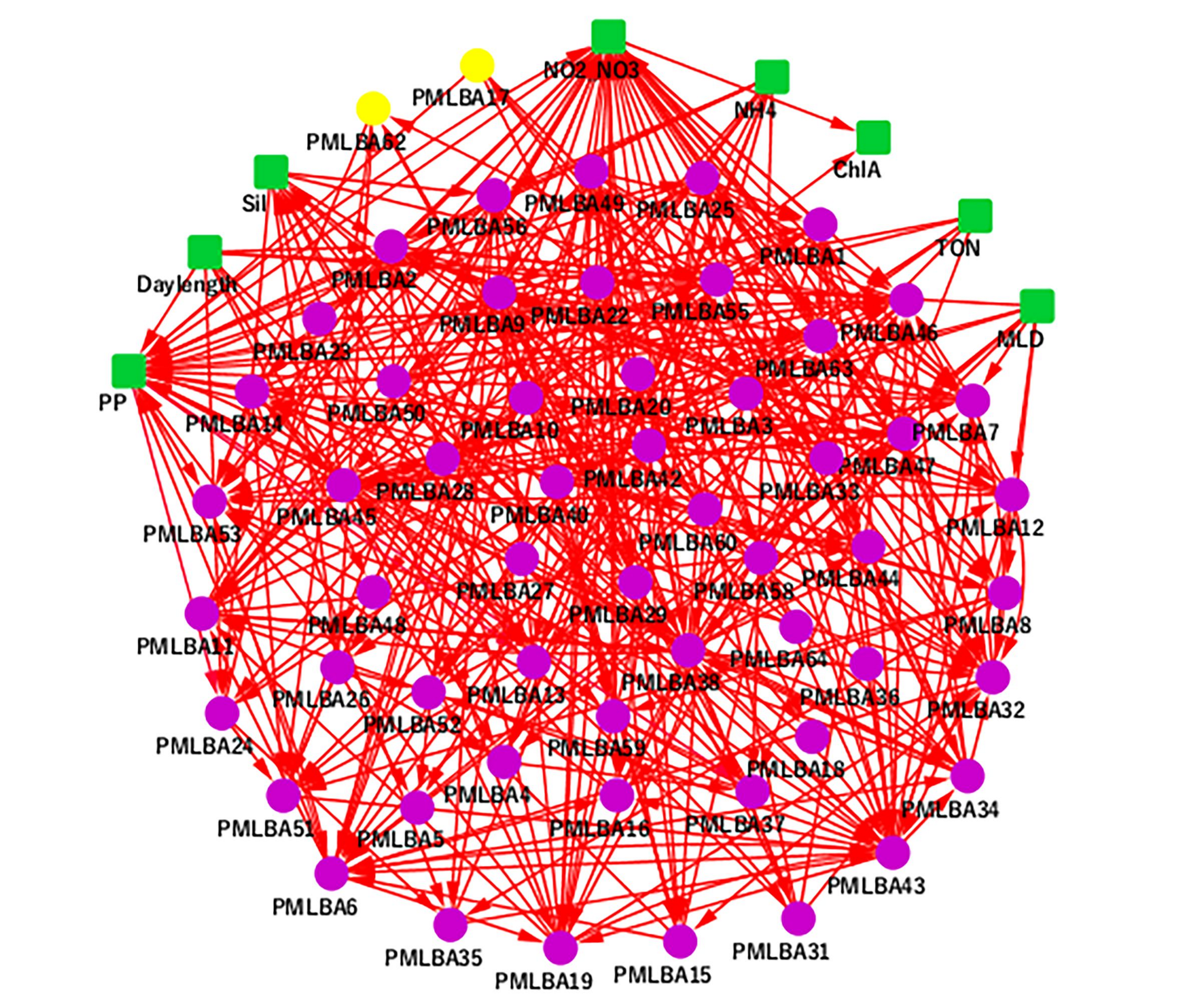

Supplement: Supplementary file 1 [file genes-10-00216-s001.zip › Supplementary/figureS1.tif]
